# Genetic Variability in Populations of the Southern Chinch Bug, *Blissus insularis*, Assessed using AFLP Analysis

**DOI:** 10.1673/031.011.17301

**Published:** 2011-12-31

**Authors:** Ambika Chandra, James A. Reinert, Jonathan LaMantia, J. Blake Pond, David R. Huff

**Affiliations:** ^1^ Texas AgriLife Research, Texas A&M System, 17360 Coit Road, Dallas TX 75252–6599; ^2^ Crop and Soil Sciences Department, Pennsylvania State University, University Park, PA 16802; ^3^ Current address, UT Southwestern Medical Center, 5323 Harry Hines Blvd. Dallas, TX 75390

**Keywords:** AMOVA, gene flow, genetic distance, St. Augustine grass

## Abstract

Southern chinch bug, *Blissus insularis* Barber (Heteroptera: Blissidae), is the most destructive insect pest of St. Augustine grass, *Stenotaphrum secundatum* Waltz (Kuntze), in the southern United States. The present study is focused on assessing genetic variability in five populations of *B. insularis* collected from Texas and Florida where St. Augustine grass is widely grown. The amplified fragment length polymorphism technique was used to DNA fingerprint individuals from each population (a total of 46 individuals) using five primer combinations (*Eco*RI*/MSe*I). Analysis of molecular variance results show no evidence to support significant genetic variability among Texas and Florida populations of *B*. *insularis.* Nearly all genetic variation was found to reside within populations (95%), with only approximately 3% residing among populations between regions. Low G_ST_ values obtained from POPGENE and low F_ST_ values obtained from the analysis of molecular variance both support the conclusion for high levels of gene flow resulting from interbreeding and/or migratory events among the populations. A Mantel test of Euclidean squared distances showed no correlation between the genetic distance and geographic distance matrices of tested populations of *B. insularis*. The results of the present study suggests that gene flow is occurring among populations of *B*. *insularis* and, therefore, breeders need to be aware that this resistance will most likely not remain localized, and it has the potential to spread as a result of migratory events.

## Introduction

Chinch bugs (Hemiptera: Blissidae: *Blissus*), *B. insularis*, are common pests of agronomic crops and turfgrasses. The four most economically important species of chinch bugs prevalent in the United States are the southern chinch bug, *B. insularis* Barber; the common chinch bugs, *B. leucopterus leucopterus*; the hairy chinch bug, *B. l. hirtus*; and the western chinch bug, *B. occiduus*. Although germplasms resistant to individual species of chinch bugs have been identified (Reinert et al. 2004), a comprehensive study conducted by Anderson et al. (2006) documented the importance of developing grass varieties that exhibit resistance to multiple species of chinch bugs for wider adaptability, reducing the need for pesticides and promoting the adoption of integrated pest management strategies.

All four economically important species of chinch bugs have been reported to have extensive and overlapping host range and geographic distributions (Anderson et al. 2006; Pierson et al. 2007; Reinert unpublished data). These overlapping ranges warrant the need to implement molecular tools, such as DNA markers, for species identification and to assess genetic structures of the genus *Blissus*. The amplified fragment length polymorphism (AFLP) marker system has been successfully used in several population genetics studies of insects (Reineke et al. 1999; Gaviria et al. 2006; Clark et al. 2007; Kazachkova et al. 2007; Krumm et al. 2008), including chinch bugs. Pierson et al. (2007) investigated the genetic variation between two species of chinch bugs, namely *B. occiduus* and *B. l. leucopterus*, using AFLP markers and showed that the majority (63%) of variability is present within species and approximately 37% variability is present between species. To our knowledge, the genetic structure of populations within and among species of chinch bugs has yet to be reported.

In the present study, the level of genetic variability in *B. insularis*, which is considered a major pest of St. Augustine grass, *Stenotaphrum secundatum* Waltz (Kuntze), throughout the southern United States was investigated. *B. insularis* has also been reported on other warm-season turfgrasses such as bermuda grass, *Cynadon dactylon;* bahia grass, Paspalum notatum; centipede grass, Eremochloa ophiuroides; and zoysia grass, *Zoysia* spp. (Kerr 1966). ‘Floratam’, a cultivar of St Augustine grass, was extensively planted in the southern United States following its release in 1973 (Horn et al. 1973) primarily because it exhibited good resistance to *B. insularis*. Resistance of ‘Floratam’ to *B. insularis* is categorized as antibiosis because of high chinch bug mortality (80% to >90% mortality within a 7-day feeding period) and low oviposition rates (Reinert and Dudeck 1974). However, after 14 years of commercial production and planting, the resistance of Floratam to *B. insularis* began to breakdown (Busey and Center 1987). Since 1987, turfgrass breeders have been striving to develop cultivar(s) with resistance to newer biotypes of *B. insularis.* The cultivars, ‘FX-10’ and ‘Captiva’, were developed and released in Florida in 1993 and 2008, respectively (Busey 1993; Nagata and Cherry 2003), and provided resistance to Florida populations of *B. insularis* (>90% mortality within 14 days) that had overcome the resistance expressed in Floratam (Busey and Center 1987; Nagata and Cherry 2003). Preliminary studies show that these three cultivars of *S. secundatum* (Floratam, FX-10,and Captiva) are susceptible to populations of *B. insularis* collected from south Texas with <20% of the chinch bugs killed by these cultivars within a 7-day feeding period in laboratory studies (Reinert et al., manuscript in preparation). Based on these herbivory studies, there appears to be differences among populations of *B. insularis*.

The objective of the present study was to assess the genetic variability between different populations of *B. insularis* using AFLP marker system. AFLP was chosen for this study because it does not require any prior knowledge of the genome (Vos et al. 1995) and generates a large number of polymorphic loci which are advantageous for studying genetic diversity and population structures (Salvato et al. 2002; Campbell et al. 2003; Blanc et al. 2006).

## Materials and Methods

### Sample collection and DNA extraction

Populations of *B. insularis* were vacuum collected using a modified Echo leaf vacuum (www.echo-usa.com) from *S. secundatum* lawns growing in three geographically different locations in Texas and two locations in Florida (Table 1). In the laboratory, a mechanical aspirator was used to separate the chinch bugs from plant material and debris. Collected chinch bugs are maintained in 95% ETOH and stored at -20° C to avoid DNA degradation. Twenty individuals were chosen randomly from each population. For DNA isolation a modified cetyltrimethylammonium bromide method was used (Black and Duteau 1997). Quantification of extracted DNA was performed to a known concentration of λ DNA by loading 1.0 µl of extracted DNA mixed with 1.0 µl of tracking dye to a 1% agarose gel, which was electrophoresed for 30 min. Ethidium bromide stained gels were visualized using the Gel Doc System.

### Amplified Polymorphic Length Polymorphism (AFLP)

The AFLP procedure was performed in three steps: 1) DNA template preparation, 2) DNA template pre-amplification, and 3) AFLP selective amplification.

**Table 1.**
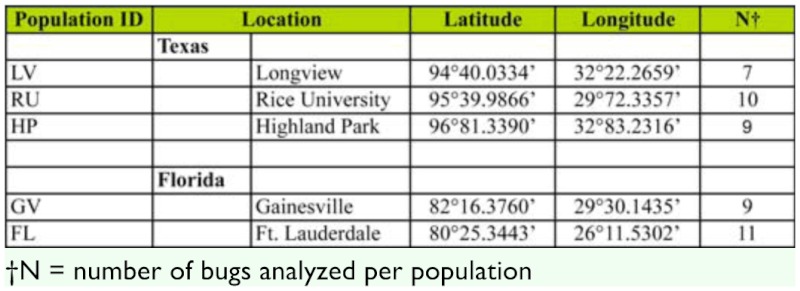
Locations of collected *B*. *insularis* populations from St. Augustine grass.

**DNA template preparation.** A total of 7 µl of genomic DNA (1.0 *µg*) was incubated with *Eco*RI and *Mse*I restriction endonucleases for 2.5 h at 37° C followed by 15 min at 70° C in a total volume of 12.5 µl of restriction digestion mixture per reaction, which contained 0.0625 µl of *Eco*RI (100,000 U/ml), 0.125 µl of MseI (50000 U/ml), 1.25 µl of 10× One-phor-all, 0.125 µl of BSA (10 mg/mL), and 3.938 µl of autoclaved nanopure water to make up the final volume. A total of 5 µl of adapter ligation mixture; consisting of 0.5 µl of 10× T4 DNA ligase buffer, 0.15 µl of T4 DNA ligase (2,000,000 U/ml), 0.5 µl of *Eco*RI adapter mix (5 µM), 0.5 µl of *Mse*I adapter mix (50 µM) (Table 2), and 3.35 µl autoclaved nanopure water per reaction; was then added into each restriction digestion mixture and incubated for 8–10 h at 25° C. After incubation, the samples were diluted with 135 µl of TE buffer and stored in a -20° C freezer. All restriction enzymes, buffers, and ligation components were supplied by New England BioLabs supplies (www.neb.com). Adaptors were synthesized by Integrated DNA Technologies (www.idtdna.com)

**Table 2.**
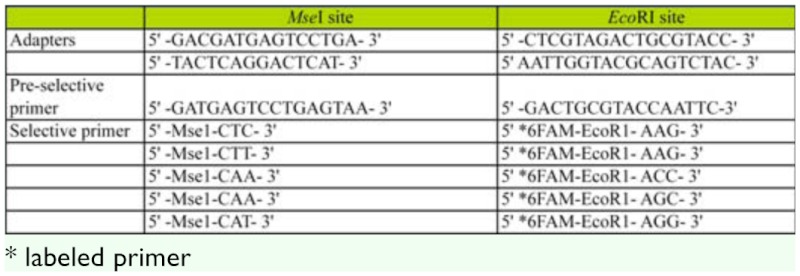
List of adaptors and selective primers used to characterize AFLP band patterns in five populations of *B. insularis.*

**Pre-amplification of DNA template.** A 22 µl master mix containing 2.5 µl 10× Taq buffer, 0.1 µl of Taq polymerase (5 U/µl), 1.5 µl of MgCl_2_ (25 mM), 0.3 µl of dNTP mix (10 mM), 0.5 µl of *Eco*RI*-*C (10 µM), 0.5 µl of *Mse*I—A (10 µM), and 16.6 µl of autoclaved nanopure water was added to 3 µl of diluted ligation product obtained from the previous step. Each reaction tube was then placed in a thermal cycler and amplified using 30 PCR cycles of 72° C for 2 min, 94° C for 30 s, 56° C for 30 s, and 72° C for 2 min followed by 60° C for 10 min. The pre-selective PCR product was diluted with 167 µl of TE buffer and stored at -20° C or immediately used in selective PCR. The oligonucleotide primers used in the pre-amplification of the DNA template are complementary to the adapter/restriction site with MseI primer containing one selective nucleotide and *Eco*RI primer containing no selective nucleotide (Table 2). All PCR components were supplied from Applied Biosystems/Life Technologies (www.appliedbiosystems.com). Primers were synthesized by Integrated DNA Technologies.

**AFLP selective amplification.** A total of 7 µl of PCR master mix was made consisting of 1.25 µl of 10× Taq buffer, 0.1 µl of Taq polymerase (5 U/ul), 1 µl of MgCl_2_ (25 mM), 0.15 µl of dNTP mix (10 mM), 1 µl of BSA (10 mg/ml), and 3.5 µl of autoclaved nanopure water per reaction. A selective primer master mix was made for each of the five primer combination (Table 2) used consisting of 2.5 µl of *Mse*I (100 µM) selective primer and 0.5 µl of 6FAM labeled *Eco*RI (100 µM) selective primer. The PCR master mix and 3 µl of the primer master mix were then mixed with 2.5 µl of pre-selective PCR products in PCR tubes. The tubes were placed in a thermal cycler and amplified using following parameters: 94° C for 2 min, 1 cycle of 94° C for 30 s, 65° C for 30 s (lowering the annealing temperature by 0.7° C per cycle), 72° C for 2 min; then 23 cycles of 94° C for 30 s, 56° C for 30 s, and 72° C for 2 min, and finally 72° C for 10 min before holding at 4° C. After selective PCR amplification was complete, the selective PCR products were stored at - 20° C or were immediately prepared for AFLP analysis. In preparation of AFLP analysis, 0.5 µl of GeneScan - 500 ROX Size Standard (ABI), 8.5 µl of Hi-Di Formamide (Applied Biosystems), and 1 µl of selective PCR product were mixed and placed in 96-well plates. The plates were analyzed using the ABI3130 capillary system.

## Data analysis

A total of 501 AFLP markers were scored for each individual bug. For each individual bug, AFLP markers were scored as Is when present and 0s when absent. Data was scored using Genographer version 2.1 using file type — Gene Mapper Filter and Sizing Algorithm — Cubic Spline Interpolator. Bands with a peak height 300 or greater and 70 bp or larger were scored. All band sizes were rounded down to the nearest whole basepair and placed in a basepair length bin. Bins with multiple bands were considered stutters and only counted once. All bins with bands were converted to 1 and all empty bins were converted to 0.

**Gene diversity and gene flow.** The resulting data was analyzed using POPGENE version 1.32 (Yeh and Boyle 1997) to assess the percent polymorphism, gene diversity estimates of total populations (H_T_) based on Nei (1973), genetic differentiation between populations (G_ST_), and gene flow estimates (Nm). Assuming Hardy-Weinberg equilibrium, the analyses were conducted at three levels: individuals, regions, and whole population using dominant marker data. H_T_ values were estimated for each individual population and G_ST_ values were estimated for populations within region and all five populations. G_ST_ is expressed as H_T_ — H_S_/H_T_ where H_T_ is gene diversity of the total population and H_s_ is gene diversity of the single population. Gene flow was estimated from G_ST_ expressed as Nm = 0.5 (1- G_ST_/ G_ST_) (McDermott and McDonald 1993).

**Analysis of molecular variance.** Analysis of molecular variance (AMOVA) (Excoffier et al. 1992; Huff et al. 1993) was conducted using Arlequin 2.0 (Schneider et al. 2000) to asses the genetic structure and genetic variability present within and among populations. Total variation of the AFLP data set was partitioned into three components, namely: among regions, among populations within a region, and within populations. AMOVA uses a permutational testing procedure to determine the significance of these three variance components. Pairwise comparisons were conducted to test genetic divergence among populations (F_ST_).

**Phylogenetic analysis.** A phylogenetic analysis was conducted using PAUP 4.0 Beta version (Swofford 1998). A neighbor joining tree was generated using PAUP based on the Euclidian squared pairwise genetic distance of the 46 individual bugs belonging to five different regions obtained using Arlequin. MEGA version 3.1 (Kumar et al. 2004) was used to view and label trees generated from PAUP 4.0. Bootstrap analysis was conducted to evaluate branch robustness of the resulting trees. Bootstrap support was estimated using 1000 bootstrap replicates, and each replicate consisted of 10 heuristic searches, random addition sequences with branch swapping, and the multree option off.

**Principle components analysis (PCoA)** Principle components analysis was conducted using NTSYSpc version 2.1 (Rohlf 2002) to construct a three-dimensional plot of the data for better visual representation.

**Mantel test.** A Mantel test (Mantel 1967) with 1000 permutations was conducted using the MxCOMP feature of the NTSYSpc version 2.1 to test the correlation between geographic distance and the pairwise genetic distance between the five sampled populations.

## Results and Discussion

Individual bugs from each population that produced banding patterns using all five primer pairs were used in the AFLP analysis. This resulted in seven individuals from the Longview, TX population; ten from the Rice University, Houston, TX population; nine from the Highland Park, TX population; nine from the Gainesville, FL population; and 11 from the Ft. Lauderdale, FL population making a total of 46 individuals analyzed in the present study (Table 1). The five primer combinations used to analyze the 46 individuals from the five populations of *B. insularis* generated a total of 501 polymorphic loci with their fragment sizes ranging from 70–495 bp. High levels of polymorphisms were observed within each population ranging from 68.66% for the Longview, TX population to 85.43% for the Highland Park, TX population (Table 3), with an average of 78.04%.

**Table 3.**
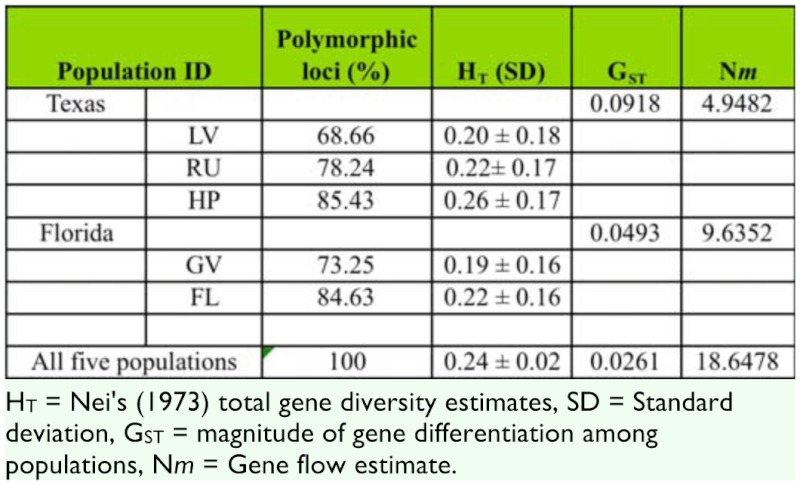
Descriptive statistical analysis of AFLP markers, across five populations of B. *insularis* belonging to two regions, using POPGENE version 1.32.

Genetic diversity, measured as population heterozygosity (H_T_) values calculated from all sub-populations, was high and ranged from 0.1898 for the Gainesville, FL population to 0.2574 for the Highland Park, TX population with an average gene diversity of 0.21844 (Table 3). Analysis across all five of the populations also revealed high genetic diversity value of 0.2428 among populations. Genetic variation within and among populations was measured as G_ST_. G_ST_ values of 1.0 indicate that a majority of genetic variability resides between populations while lower G_ST_ values (<0.5) indicate that a majority of the genetic variability resides within a given population. In the present study, G_ST_ value among all sampled populations was low (0.0261) indicating a high degree of genetic variability within population (97.4%) and low variation among populations (2.61%) (Table 3). These results indicate that there is apparently little genetic differentiation among the sampled populations. The lack of genetic differentiation among populations is generally considered the result of sufficient gene flow, typically in the form of migration, occurring across populations to counteract any effects of selection and/or genetic drift.

Gene flow is commonly measured as N*m* where N is the number of individuals in a population and M is the proportion of individuals in the population as a result of immigration. Nm values >1 indicate a high level of gene flow and that the effects of gene flow on population differentiation is greater than the effect of random genetic drift (Bossart and Prowell 1998). In the present study, N*m* values were high in two tested regions, Texas and Florida, (Table 3) suggesting high gene flow from events such as interbreeding and migration. High gene flow values also suggest that populations among species will become genetically homogeneous in the absence of counteracting forces such as strong differential selection.

**Table 4.**
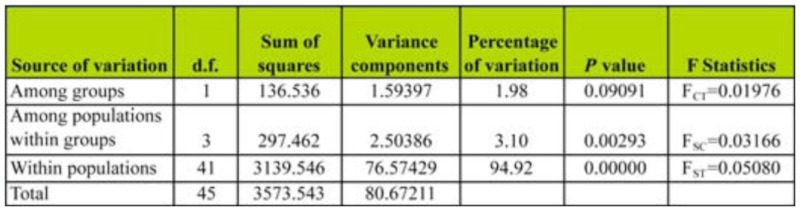
Analysis of molecular variance (AMOVA) for five populations of *B.* *insularis* using Arlequin version 2.0.

**Figure 1.**
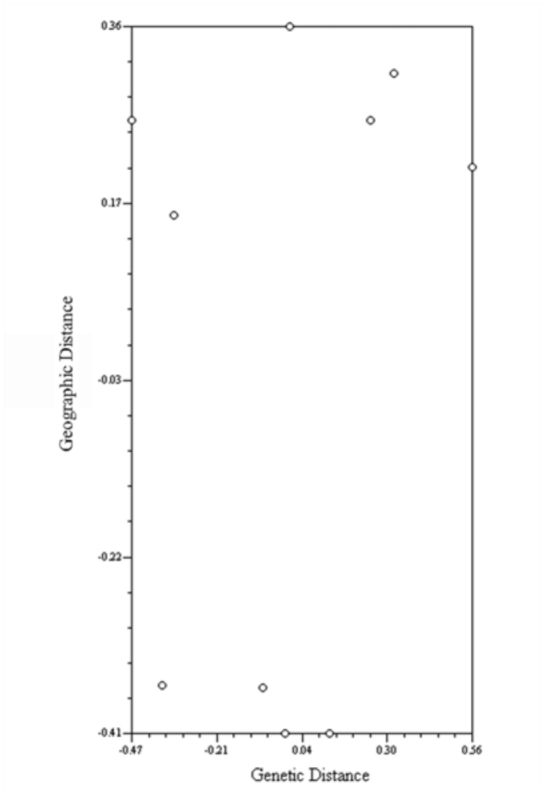
Mantel test (N= 10; r = 0.22021 ;*P* = 0.7682) of genetic distance versus geographic distance indicating lack of correlation. High quality figures are available online

AMOVA showed that the difference among groups was not significant (*P* = 0.09091), but that the amounts of genetic variation among populations within groups and within populations were significant at *P* = 0.00293 and 0.0000, respectively (Table 4). AMOVA showed that approximately 95% of the variation in the data set was from genotypic variation within populations. Only 3% of the variation was attributed to differences among populations within regions while the remaining 2% was due to the variation among regions (Table 4). A Mantel test (Figure 1) showed that the genetic and geographic distances matrices were not significantly correlated (N = 10; r = 0.22021; *P* = 0.7682) indicating that there was no genetic isolation in regions of southern chinch bug populations tested in this study. Genetic divergence (F_ST_) values less than 0.2 indicates high gene flow and in the present study F_ST_ for five subpopulations was 0.05080 indicating low differentiation among populations (Table 4). These results are consistent with those obtained from POPGENE (Table 3). This low level of differentiation between populations supports the possibility of gene flow among all the populations examined.

**Figure 2.**
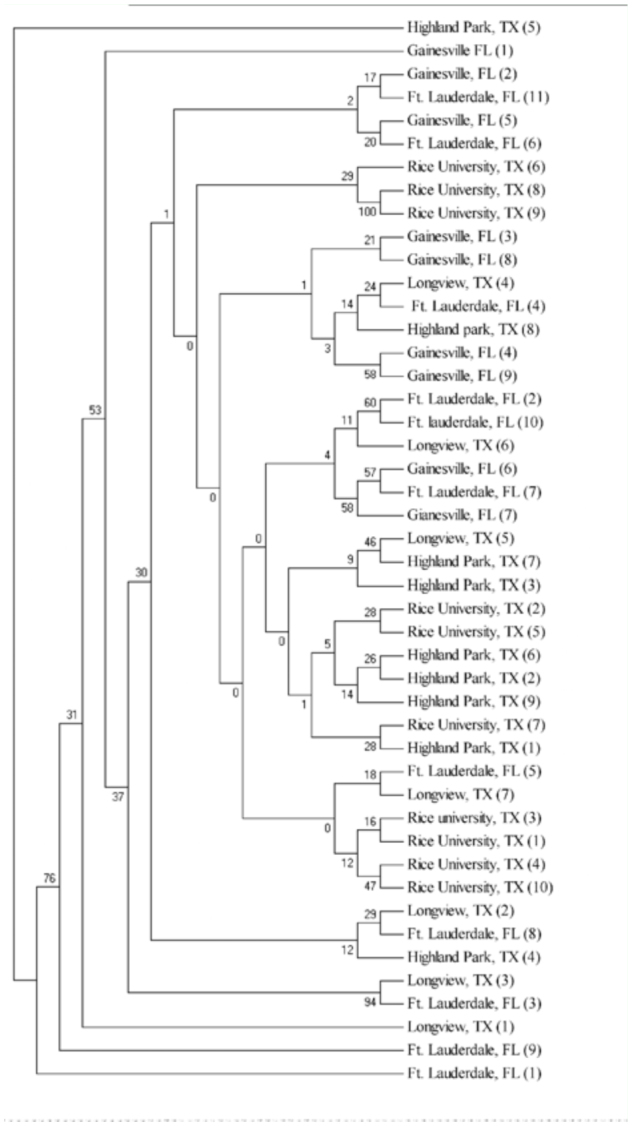
A phylogenetic neighbor joining tree based on euclidian squared pairwise genetic distance generated using PAUP 4.0 Beta. 46 *B.* *insularis* belonging to five different regions included in the tree. Underlined samples are from the Longview, TX population. Boostrap values are indicated above branch. Number in Parenthesis indicates the number for the bug sample analyzed in each population. High quality figures are available online

**Figure 3.**
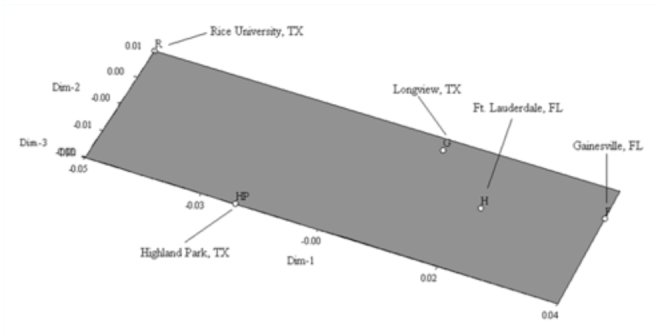
Principle coordinate analysis based on mean pairwise genetic distance for five different population of B. insularis using NTSYS pc22. High quality figures are available online

As expected, a phylogenetic neighbor-joining tree based on Euclidian-squared, pairwise genetic distance of 46 samples of *B. insularis* (Figure 2) shows no clearly defined genetic structure among regions. However, the Longview, TX population (underlined in Figure 2) appeared to be a particularly unique population where individuals from within this population tend to cluster with individuals from the Florida populations. This scenario is more apparent when principle component analysis was conducted and a three-dimensional Eigen plot (Figure 3) was graphed, which depicts mean pairwise genetic distance for the five populations of *B. insularis.* This situation leads us to speculate that the Longview, TX population is likely leading to the non-significant genetic variability among regions in our AMOVA analysis. Therefore, the next step was to conduct an AMOVA analysis without the inclusion of the Longview, TX population. However, when an AMOVA was conducted without the inclusion of the Longview, TX population, the difference among groups was again non-significant (*P* = 0.36950) and the difference in genetic variation among populations within groups and within populations were both significant at *P* = 0.03226 and 0.0000, respectively. Without the inclusion of the Longview, TX population, the AMOVA showed that 93.4% of the variation in the data set was from genotypic variation within populations, 2.4% of the variation was attributed to differences among populations within regions, while the remaining 4.2% was due to the variation among regions. Therefore, an AMOVA either with or without the inclusion of the Longview, TX population gave similar results, in that, there was no significant genetic variability present among regions, and that the majority of the genetic AFLP variability measured was found to be present within populations.

Based on the results of the present study the resistance mechanisms of *S. secundatum* to *B. insularis* do not appear to be related to specific population differences in *B. insularis,* but rather it is more likely that such resistance results from the expression and regulation of specific genes from individual bugs. The present study also suggests that gene flow is occurring among populations of *B. insularis* and, therefore, breeders need to be aware that this resistance will most likely not remain localized and it has the potential to spread as a result of migratory events. In the future, a more detailed investigation might be performed by increasing the number of populations of *B. insularis* from the geographic distribution of *S. secundatum;* especially - a few more populations collected and analyzed from the Longview, TX region might be informative and useful in better understanding the population genetic structure of *B. insularis.*

## **Abbreviations**

AFLP: amplified fragment length polymorphism
AMOVA: analysis of molecular variance
F_ST_: genetic divergence
G_ST_: total gene diversity estimates
Nm: gene flow estimates
PCoA: principle component analysis
